# ﻿Phylogenetic assessment and taxonomic revision of *Scytalidium* (*Helotiales*, *Leotiomycetes*)

**DOI:** 10.3897/imafungus.16.164608

**Published:** 2025-10-14

**Authors:** Shuo-Qiu Tong, Yi-Fan Yang, Peng Li, Yong-Jun Wu, Bing-Da Sun, Zhi-Yuan Zhang

**Affiliations:** 1 College of Life Sciences, Institute of Agro-bioengineering, Guizhou University, Guiyang 550025, China Guizhou University Guiyang China; 2 China General Microbiological Culture Collection Center, Institute of Microbiology, Chinese Academy of Sciences, Beijing 100101, China Chinese Academy of Sciences Beijing China; 3 College of Eco-Environmental Engineering, Guizhou Minzu University, Guiyang 550025, China Guizhou Minzu University Guiyang China

**Keywords:** *
Leotiomycetes
*, molecular systematics, re-classification, taxonomy, three new taxa

## Abstract

Members of *Scytalidium* are primarily saprotrophic and are known for their ability to colonize a variety of substrates, including soil, decaying plant material, and wood. During an investigation of soil microfungi in *Capsicum annuum* cultivation areas of China, seven *Scytalidium* isolates were obtained from soil samples collected in Guizhou. In this study, we revised the genus *Scytalidium* by combining morphological characteristics and phylogenetic analyses based on concatenated ITS–LSU sequences. The results showed that *Scytalidium* sensu stricto comprises 16 species, including the type species *S. lignicola* and four novel species proposed in this study (*S. chlamydosporum*, *S. guizhouense*, *S. rodionovae*, and *S. tongrenense*). Ten species were excluded, and six species were treated as uncertain due to the lack of available molecular sequences. This study revised the genus *Scytalidium*, expanded its species diversity and geographical distribution, and lays the foundation for future taxonomic research on this genus.

## ﻿Introduction

Pesante (1957) introduced the genus *Scytalidium*, with *Scytalidium lignicola* as the type species. This genus is characterized by the possession of two types of arthroconidia: hyaline, thin-walled, cylindrical entities formed by the fragmentation of undifferentiated hyphae, and brown, thick-walled, broadly ellipsoid entities borne in an intercalary fashion (i.e., chlamydospore-like cells) (Pesante 1957). Currently, there are 34 records of *Scytalidium* in Index Fungorum (https://www.indexfungorum.org, accessed July 2025). Many former *Scytalidium* species have been transferred to other genera, with several even serving as type species for newly established genera; for example, *Acidomyces*, with *S. acidophilum* as type (Selbmann et al. 2008); *Neoscytalidium*, typified by *S. dimidiatum* (Crous et al. 2006), later superseded by *S. hyalinum* as type and synonym of *N. dimidiatum* (Phillips et al. 2013); and *Mycothermus*, based on the reclassified *Torula thermophila* (invalidly proposed by Natvig et al. 2015; validated by Wang et al. 2019). Additionally, S. aurantiacum var. album is recognized as a synonym of *S. album* (https://www.indexfungorum.org, https://www.mycobank.org). To date, there are still 29 species remaining in *Scytalidium*. Among them, *S. fulvum*, *S. hepiali*, *S. melanoxylicola*, *S. nielamuense*, *S. verruculosum*, and *S. xigazense* lack available molecular sequence data (Morgan-Jones et al. 1984; Li and Sun 1988; Wu and Zhang 2010; Awasthi et al. 2020).

The genus *Scytalidium* exhibits a broad ecological distribution, being found in a wide range of environments and substrates. Several species are primarily associated with plants, including wood and timber, where they act as saprobes, cause blue staining, or decay (e.g., *S. album*, *S. aurantiacum*, *S. circinatum*, *S. cuboideum*, and *S. sphaerosporum*) (Klingström and Beyer 1965; Sigler and Wang 1990; Robinson et al. 2007), or as pathogens (*S. lignicola* on cassava (*Manihot esculenta*)) (Oren et al. 2001; Kang et al. 2010; Silva et al. 2013; De Medeiros et al. 2019). Some are also reported from dead plant material such as twigs (e.g., *S. synnematicum*) (Crous et al. 2023). Other species are mycoparasitic and known from cultivated fungi, including *S. auriculariicola* on *Auricularia polytricha* and *S. ganodermophthorum* on *Ganoderma lucidum* (Kang et al. 2010; Peng et al. 2014). Soil represents another common habitat, with several species exhibiting saprophytic lifestyles, often isolated from agricultural or forest soils (e.g., *S. chlamydosporum*, *S. guizhouense*, *S. tongrenense*, and *S. terrigenum*) (Jeong et al. 2025). Additionally, one species (*S. assmuthi*) has been isolated from the gut of a wood-feeding termite, suggesting an association with insects (Manawasinghe et al. 2024). Another species (*S. philadelphianum*) has been recovered from compressed air, suggesting occurrence in airborne or industrial environments (Crous et al. 2022).

The chili pepper (*Capsicum annuum*) is a globally grown and consumed spice crop that is rich in vitamins. It has a long history of cultivation in China. During our investigation of soil microfungi in *C. annuum* cultivation areas of Guizhou, China from 2022–2024, we obtained seven isolates of the genus *Scytalidium*. This study has the following objectives: (1) to describe three novel *Scytalidium* species collected from soil; (2) to propose *S. rodionovae* as a new species, with strain 3C designated as the ex-type culture, to legitimize the taxon; (3) to provide a checklist that includes substrate, availability of molecular data, morphological characteristics, and country of origin; and (4) to revise the genus *Scytalidium* by combining morphological characteristics and phylogenetic analyses based on concatenated ITS–LSU sequences.

## ﻿Materials and methods

### ﻿Fungal isolation and morphological characterization

Soil sampling was conducted annually in July from 2022–2024 across three *Capsicum annuum*–growing regions in Guizhou Province: Liuguang Town (116.44E, 26.99N; Xiuwen County, Guiyang), Shiban Town (106.65E, 26.46N; Huaxi District, Guiyang), and Qiaojia Town (108.44E, 28.28 N; Yanhe County, Tongren). Soil samples were collected using a shovel at a depth of 0–10 cm from the soil surface, stored in sterile Ziploc bags, transported to the laboratory under refrigeration (4 °C), and processed immediately upon arrival. Fungi were isolated using the dilution method described in Tong et al. (2023).

After obtaining pure colonies on potato dextrose agar (PDA; Coolaber, China), they were transferred onto fresh PDA and synthetic nutrient-poor agar (SNA; Coolaber, China) media, followed by incubation at 25 °C in darkness for 14 d to observe macroscopic and morphological characteristics of the colonies. Color names and codes adhered to the *Methuen Handbook of Colour* (Kornerup and Wanscher 1978). For light microscopic observations, slides were prepared from cultures grown on PDA, and 25% lactic acid was used as the mounting fluid. Morphological features were observed and recorded using a Zeiss Axio Imager A2 microscope and a Zeiss AxioCam MRc color digital camera (Carl Zeiss Ltd., München, Germany). Structural measurements were carried out using Digimizer software (v6.4.5), with a minimum of 50 measurements taken for each structure, such as conidia.

The ex-type strains for the novel species were deposited at the China General Microbiological Culture Collection Center (CGMCC), China. Additionally, all living cultures were stored in a metabolically inactive state (i.e., kept in sterile 30% glycerol in a –80 °C freezer) and were deposited in the College of Eco-Environmental Engineering, Guizhou Minzu University, and at the Institute of Agro-bioengineering, Guizhou University, China. Dried culture (at 50 °C) specimens were deposited at the Fungarium (HMAS), Institute of Microbiology, Chinese Academy of Sciences (CAS). MycoBank numbers were registered for new names, including novel species and combinations.

### ﻿DNA extraction, PCR amplification, and sequencing

Scraped fungal mycelia were used for DNA extraction with a BioTeke Fungal Genomic DNA Extraction Kit (DP2032, BioTeke, Beijing, China), following the manufacturer’s protocol. Polymerase chain reactions (PCR) were performed using internal transcribed spacer (ITS) and large nuclear ribosomal subunit rDNA (LSU) regions, which were amplified using primer pairs ITS1/ITS4 (White et al. 1990) and LR0R/LR5 (Vilgalys and Hester 1990), respectively. PCR was carried out in a 25 μL reaction volume containing 12.5 μL of 2× Power Taq PCR MasterMix (a premixed and ready-to-use solution including 0.1 units/μL Taq DNA Polymerase, 500 μM dNTP mixture each [dATP, dCTP, dGTP, dTTP], 20 mM Tris-HCl pH 8.3, 100 mM KCl, 3 mM MgCl2, stabilizer, and enhancer), 1 μL of each primer, 1 μL genomic DNA extract, and 8.5 μL double-distilled water. The PCR products were purified and sequenced at Shanghai Sangon Biological Engineering Technology & Services Co., Shanghai, China. Raw forward and reverse sequences were assembled using Lasergene software (version 6.0, DNASTAR). All newly generated sequences have been deposited in the GenBank database (Table 1).

**Table 1.** Strains/vouchers used in this study, with information on the GenBank accessions of the sequences.

Species	Strain/voucher	GenBank accession no.		References
		ITS	LSU	
*Albonectria rigidiuscula*	CBS 133754	MW827602	MW827641	Crous et al. 2021
*Bisifusarium dimerum*	CBS 108944 T	JQ434586	JQ434514	Ropars et al. 2012
*Caliciopsis calicioides*	211	JX968549	NA	Assefa et al. 2014
*Caliciopsis moriondi*	CBS 146717	MN156540	NA	Migliorini et al. 2020
*Caliciopsis orientalis*	CBS 138.64	KP881690	MH870024	Wood et al. 2016; Vu et al. 2019
*Caliciopsis pinea*	CBS 139.64	KP881691	NA	Wood et al. 2016
*Cyanonectria multiformsporum*	CGMCC 3.20774 T	OL897004	OL897046	Zhang et al. 2023a
*Fusarium callistephi*	CBS 187.53 T	MH857158	MH868694	Vu et al. 2019
*Fusarium hoodiae*	CBS 132474 T	MH866022	MH877470	Vu et al. 2019
*Fusarium oxysporum*	LC13766	MW016600	NA	Wang et al. 2022
*Geejayessia celtidicola*	CBS 125502 T	HM626657	HM626669	Schroers et al. 2011
*Geniculospora grandis*	CBS 261.84	MH861735	MH873440	Vu et al. 2019
*Hyaloscypha hepaticicola*	CBS 652.89 T	MH862193	MH873881	Vu et al. 2019
*Hypoxylon florendophyticum*	GUCC 193025.1 T	ON791190	ON791224	Zhang et al. 2023b
*Hypoxylon hinnuleum*	MUCL 3621 T	MK287537	MK287549	Sir et al. 2019
*Hypoxylon investiens*	CBS 118183	KC968925	KY610450	Kuhnert et al. 2014; Wendt et al. 2018
*Hypoxylon lateripigmentum*	MUCL 53304 T	KC968933	KY610486	Kuhnert et al. 2014; Wendt et al. 2018
*Hypoxylon lignicola*	MFLUCC 16-0926 T	MK828609	MK835808	Luo et al. 2019
*Lasiobelonium lonicerae*	FC-2270	AB481284	AB481319	Hosoya et al. 2010
*Luteonectria albida*	CBS 102683	MW827615	MH874402	Crous et al. 2021; Vu et al. 2019
*Monochaetia dimorphospora*	NBRC 9980 T	LC146750	LC146750	Liu et al. 2019
*Monochaetia hanzhongensis*	CFCC 54451 T	OK339776	OK339747	Jiang et al. 2023
*Monochaetia quercus*	CBS 144034 T	MH554171	MH554365	Liu et al. 2019
*Mycofalcella calcarata*	CCM F-10289	KC834065	KC834033	Baschien et al. 2013
*Mycothermus thermophiloides*	CBS 183.81 T	LT993603	LT993603	Wang et al. 2019
*Mycothermus thermophilus*	CBS 625.91 T	LT993604	LT993604	Wang et al. 2019
*Neocosmospora regularis*	CBS 230.34 T	LR583763	LR583967	Sandoval-Denis et al. 2019
*Neocosmospora silvicola*	CBS 123846 T	LR583766	LR583971	Sandoval-Denis et al. 2019
*Neodevriesia cladophorae*	CGMCC 3.17901 T	KU578112	KU578114	Wang et al. 2017
*Neodevriesia knoxdaviesii*	CBS 122898 T	MH863254	MH874778	Vu et al. 2019
*Neodevriesia metrosideri*	CBS 145084 T	NR_161141	NG_066296	Crous 2018
*Neodevriesia strelitziae*	CBS 122379 T	MH863206	EU436763	Vu et al. 2019; Arzanlou and Crous 2008
*Neoscytalidium hyalinum*	CBS 145.78 eiT	MH861121	DQ377922	Vu et al. 2019; Crous et al. 2006
*Neoscytalidium hylocereum*	PSU-HP01 T	LC590859	NA	Wonglom et al. 2023
*Neoscytalidium novaehollandiae*	CBS 122071 T	MH863173	MH874720	Vu et al. 2019
*Nothofusarium devonianum*	NRRL 22134 T	MW827632	MW827673	Crous et al. 2021
*Rectifusarium ventricosum*	CBS 748.79 T	HQ897816	KM231658	Gräfenhan et al. 2011; Lombard et al. 2015
*Remersonia tenuis*	CBS 784.85 T	LT993609	LT993609	Wang et al. 2019
*Remersonia thermophila*	CBS 643.91	LT993610	LT993610	Wang et al. 2019
*Scytalidium album*	173	MF992676	MF966378	Pavlov et al. 2018
*Scytalidium album*	CBS 373.65	MH858618	MH870258	Vu et al. 2019
*Scytalidium album*	CBS 372.65 T	MH858617	MH858617	Vu et al. 2019
*Scytalidium assmuthi*	PYCC 9837 T	OR415883	OR415885	Manawasinghe et al. 2024
*Scytalidium aurantiacum*	CBS 374.65 T	MH858619	MH870259	Vu et al. 2019
*Scytalidium auriculariicola*	YBI-3 T	GU591724	NA	Peng et al. 2014
*Scytalidium candidum*	3C T	MF992675	MG018250	Pavlov et al. 2018
***Scytalidium chlamydosporum***	**CGMCC 3.28993 T**	**PV890025**	**PV890032**	**This study**
***Scytalidium chlamydosporum***	**SQT11**	**PV890026**	**PV890033**	**This study**
***Scytalidium chlamydosporum***	**SQT12**	**PV890027**	**PV890034**	**This study**
*Scytalidium chinense*	H1091 T	HQ213805	HQ221579	Geng et al. 2016
*Scytalidium circinatum*	CBS 654.89 T	NR_160180	MH873882	Vu et al. 2019
*Scytalidium circinatum*	SM14-27-6-5	MN905823	NA	Held et al. 2020
*Scytalidium cuboideum*	KACC 41223 T	GQ272628	NA	Kang et al. 2010
*Scytalidium cuboideum*	UAMH 3792	GQ503338	NA	Kang et al. 2010
*Scytalidium cuboideum*	CBS 241.62	MH858144	MH858144	Vu et al. 2019
*Scytalidium flavobrunneum*	CBS 244.59 T	MH857854	MH857854	Vu et al. 2019
*Scytalidium ganodermophthorum*	TPML 97003	GQ272622	NA	Kang et al. 2010
*Scytalidium ganodermophthorum*	UAMH 10320 T	GQ272617	NA	Kang et al. 2010
*Scytalidium ganodermophthorum*	H123	GQ272620	NA	Kang et al. 2010
***Scytalidium guizhouense***	**CGMCC 3.28992 T**	**PV890023**	**PV890030**	**This study**
***Scytalidium guizhouense***	**SQT09**	**PV890024**	**PV890031**	**This study**
*Scytalidium indonesiacum*	CBS 259.81 T	MH861338	MH873098	Vu et al. 2019
*Scytalidium infestans*	CBS 161.91 T	MH862246	MH862246	Vu et al. 2019
*Scytalidium japonicum*	CBS 494.88 T	MH873833	MH873833	Vu et al. 2019
*Scytalidium japonicum*	CBS 125804	MH863771	NA	Vu et al. 2019
*Scytalidium lignicola*	UAMH 1502 T	NR_121314	NA	Schoch et al. 2014
*Scytalidium lignicola*	CBS 125602	MH863583	NA	Vu et al. 2019
*Scytalidium multiseptatum*	CBS 693.70	MH859908	MH871702	Vu et al. 2019
*Scytalidium multiseptatum*	CBS 241.68	MH859124	MH870836	Vu et al. 2019
*Scytalidium parasiticum*	AAX0113 T	KF925449	NA	Goh et al. 2015
*Scytalidium philadelphianum*	CPC 40793 T	ON811538	NA	Crous et al. 2022
*Scytalidium sphaerosporum*	CBS 187.69	GQ272623	MG018251	Kang et al. 2010; Pavlov et al. 2018
*Scytalidium sphaerosporum*	ATCC 34392 T	NR_145360	NA	Kang et al. 2010
*Scytalidium sphaerosporum*	KACC 41222	GQ272626	NA	Kang et al. 2010
*Scytalidium synnematicum*	CCMB 207/13 T	OQ430525	OQ430526	Crous et al. 2023
*Scytalidium terminale*	CBS 171.40 T	MH856079	NA	Vu et al. 2019
*Scytalidium terrigenum*	KNUF-23-236 T	LC859329	LC859330	Jeong et al. 2025
*Scytalidium tibetense*	H1127 T	HQ213808	HQ221582	Geng et al. 2016
***Scytalidium tongrenense***	**CGMCC 3.28994 T**	**PV890028**	**PV890035**	**This study**
***Scytalidium tongrenense***	**SQT14**	**PV890029**	**PV890036**	**This study**
*Scytalidium tuberculatum*	H1195 T	HQ213809	HQ221583	Geng et al. 2016
*Scytalidium uredinicola*	CBS 578.75	MH860954	NA	Vu et al. 2019
*Setofusarium setosum*	CBS 635.92 T	MW827634	MW827675	Crous et al. 2021
*Trichopeziza sulphurea*	KUS-F52218	JN033398	JN086701	Han et al. 2014
*Tricladium obesum*	CCM F-14598	KC834068	KC834035	Baschien et al. 2013
*Geoglossum azoricum*	AMI-SPL1247	OQ618223	OQ618224	Crous et al. 2023
*Geoglossum dunense*	TUR-A 199830 T	KP744516	KP744517	Loizides et al. 2015

Notes: T: ex-type; eiT: ex-isotype; *Scytalidium vaccinii* is a synonym of *Hyaloscypha hepaticicola*, and CBS 652.89 is the type strain of *S. vaccinii*. **CBS**: Westerdijk Fungal Biodiversity Institute, Utrecht, Netherlands; **CGMCC**: China General Microbiological Culture Collection Center, Beijing, China; **CPC**: Collection of Pedro Crous housed at CBS; **MFLUCC**: Mae Fah Luang University Culture Collection, Chiang Rai, Thailand; **GUCC**: Culture Collection of the Department of Plant Pathology, Agriculture College, Guizhou University, China; **ATCC**: American Type Culture Collection, Manassas, USA; **KACC**: Korean Agricultural Culture Collection, Republic of Korea; **UAMH**: University of Alberta Mold Herbarium and Culture Collection, Edmonton, Canada; **NRRL**: Agricultural Research Service Culture Collection, National Center for Agricultural Utilization Research, USA; **NBRC**: Biological Resource Center, National Institute of Technology and Evaluation, Tokyo, Japan; **MUCL**: Université Catholique de Louvain, Louvain-la-Neuve, Belgium. Other acronyms represent personal collections; NA, not available. DNA sequences for the new isolates were in bold.

### ﻿Phylogenetic analyses

Based on BLASTn search results and recently published data (Table 1), reference sequences were downloaded and aligned using MAFFT v7.037 (Katoh and Standley 2013) and further refined with MEGA 6.06 (Tamura et al. 2013). Phylogenetic analyses of combined aligned ITS and LSU sequences were performed with Bayesian and maximum-likelihood algorithms. Maximum likelihood (ML) analysis was carried out using IQ-TREE v1.6.11 (Nguyen et al. 2015) with 10,000 bootstrap tests and the ultrafast algorithm (Minh et al. 2013). The best evolutionary models for phylogenetic analyses were selected independently for each locus using ModelFinder (Kalyaanamoorthy et al. 2017) under the corrected Akaike Information Criterion (AICc). Bayesian analyses were performed with MrBayes v3.2 (Ronquist et al. 2012). The Markov Chain Monte Carlo (MCMC) method was used to perform 5 × 10^7^ simulations with a sampling frequency of 10³ generations and a 25% burn-in. After the analysis was finished, Tracer v1.5 (Drummond and Rambaut 2007) was used to determine burn-in and confirm that both runs had converged.

## ﻿Results

### ﻿Phylogenetic analyses

**Figure 1.**
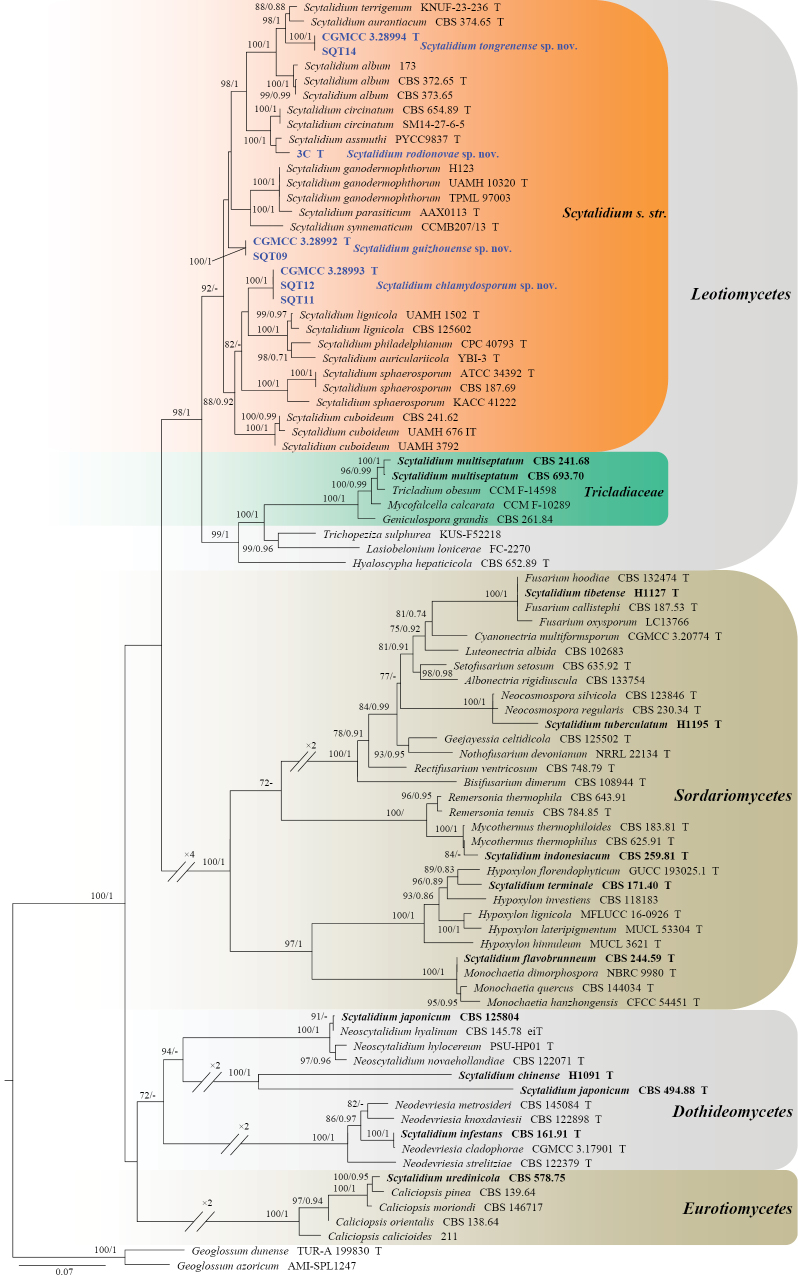
Phylogenetic tree inferred from a maximum likelihood analysis based on a concatenated alignment of ITS and LSU sequences from 87 isolates representing *Scytalidium*, related taxa, and outgroup taxa. Numbers at branches indicate support values (IQ-TREE-BS/BI-PP) above 70%/0.90. The new species are printed in bold blue, and the taxa transferred out of *Scytalidium* in bold black. Strains with a type status are indicated with “T.” The tree is rooted to *Geoglossum azoricum* (AMI-SPL1247) and *G. dunense* (TUR-A 199830).

**Figure 2.**
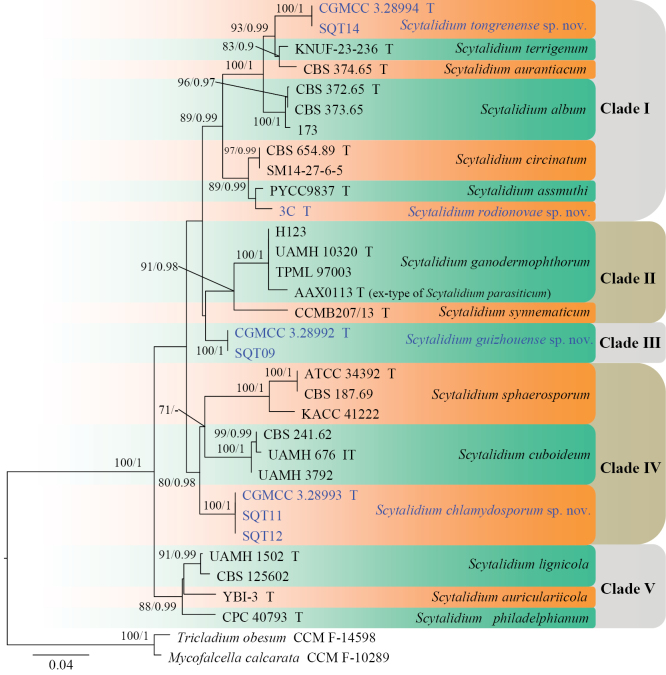
Phylogenetic tree inferred from a maximum likelihood analysis based on a concatenated alignment of ITS and LSU sequences from 33 isolates representing *Scytalidium* and outgroup taxa. Numbers at branches indicate support values (IQ-TREE-BS/BI-PP) above 70%/0.90. The new species are printed in blue. Strains with a type status are indicated with “T.” The tree is rooted to *Tricladium obesum* (CCM F-14598) and *Mycofalcella calcarata* (CCM F-10289).

Phylogenetic trees were generated to determine the class-level placement of the *Scytalidium* isolates (Analysis 1) and to resolve the phylogenetic relationships among species within *Scytalidium* sensu stricto (Analysis 2) (Figs 1, 2). *Geoglossum azoricum* (AMI-SPL1247) and *G. dunense* (TUR-A 199830) were used as the outgroups in Analysis 1, whereas *Tricladium obesum* (CCM F-14598) and *Mycofalcella calcarata* (CCM F-10289) were used as the outgroups in Analysis 2. The concatenated sequences of Analyses 1 and 2 included 87 and 33 taxa, respectively, and consisted of 1,347 characters (ITS: 542 bp and LSU: 805 bp) and 1,400 characters (ITS: 581 bp and LSU: 819 bp), respectively, with gaps.

**Analysis 1.** The phylogenetic tree showed that 16 species of *Scytalidium*, including the type species *S. lignicola* and the three new species proposed here, formed a highly supported clade (98% BS/1 PP) within *Leotiomycetes* (Fig. 1). In addition, multiple species or strains of *Scytalidium* were dispersed across *Sordariomycetes*, *Dothideomycetes*, and *Eurotiomycetes* (Fig. 1). These findings strongly suggest that *Scytalidium* is polyphyletic, with several species placed outside the main clade containing the type species, including taxa in three other classes (*Dothideomycetes*, *Eurotiomycetes*, and *Sordariomycetes*).

**Analysis 2.** The phylogenetic tree showed that *Scytalidium* formed a strongly supported clade (100/1), which can be broadly divided into five clades (Fig. 2). Clade I comprised *S. album*, *S. assmuthi*, *S. aurantiacum*, *S. rodionovae*, *S. circinatum*, *S. terrigenum*, and *S. tongrenense* with high support (89/0.99). Clade II comprised *S. ganodermophthorum* and *S. synnematicum* with high support (91/0.98). Clade III comprised only *S. guizhouense*. Clade IV comprised *S. chlamydosporum*, *S. cuboideum*, and *S. sphaerosporum* with high support (80/0.98). Clade V comprised *S. auriculariicola*, *S. lignicola*, and *S. philadelphianum* with high support (88/0.99). Additionally, the novel species proposed in this study each formed distinct subclades with strong statistical support (100/1).

### ﻿Taxonomy

#### ﻿ Scytalidium

Taxon classificationAnimaliaHelotialesChaetomiaceae

Pesante

BBE71524-BD0B-524E-B031-FDE4A1B584F4

##### Type species.

*Scytalidium lignicola* Pesante.

##### Notes.

In this study, the phylogeny of *Scytalidium* was reconstructed using concatenated ITS and LSU sequences from (i) the ex-type strain of *S. lignicola* (type species of the genus), (ii) ex-type strains of 24 additional *Scytalidium* species, and (iii) ex-type strains of phylogenetically related fungi in *Leotiomycetes*, *Sordariomycetes*, *Dothideomycetes*, and *Eurotiomycetes*, as identified through preliminary phylogenetic analyses. The results showed that *Scytalidium* is a polyphyletic genus spread across at least four classes in *Ascomycota* (Fig. 1). We formally propose to restrict *Scytalidiumsensu stricto* to a monophyletic group of 16 species, with the exclusion of all phylogenetically divergent lineages currently classified in *Scytalidium*.

### ﻿Accepted species

#### 1.﻿ Scytalidium album

Taxon classificationAnimaliaHelotialesChaetomiaceae

L. Beyer & Klingström, Svensk bot. Tidskr. 59: 35 (1965)

16BD011E-DED4-5453-BC24-7752EA408E24

##### Description and illustration.

Klingström and Beyer (1965).

##### Notes.

*Scytalidium album* was initially isolated from Norway spruce wood damaged by root rot, and it induces blue staining in timber (Klingström and Beyer 1965). *Scytalidium album* is phylogenetically closely related to *S. assmuthi*, *S. aurantiacum*, *S. rodionovae*, *S. circinatum*, *S. terrigenum*, and *S. tongrenense* (Figs 1, 2). Morphologically, *S. album* is distinguished from *S. assmuthi*, *S. rodionovae*, and *S. tongrenense* by its unknown sexual morph and simultaneous production of arthroconidia and chlamydospore-like cells (Klingström and Beyer 1965; Pavlov et al. 2018; Manawasinghe et al. 2024). *Scytalidium album* differs from *S. terrigenum* by its hyaline arthroconidia (Jeong et al. 2025) and from *S. circinatum* by the shape and size of its chlamydospore-like cells (globose or ellipsoidal, 6.4–14.4 × 4.8–9.6 μm in *S. album* vs. globose, lobed, or irregular, 4–9 × 3–9 μm in *S. circinatum*) (Sigler and Wang 1990). Furthermore, *S. album* differs from *S. aurantiacum* by its secretion of a pale-yellow pigment (Klingström and Beyer 1965). Additionally, they can be distinguished by their low sequence similarities. Based on a pairwise comparison of ITS and LSU, *S. album* (ex-type CBS 372.65) differs from *S. assmuthi* (ex-type PYCC 9837) by 7.4% (34/460 bp, four gaps) in the ITS and 4.2% (23/536 bp, one gap) in the LSU; from *S. aurantiacum* (ex-type CBS 374.65) by 2.9% (15/521 bp, no gap) in the ITS and 2.5% (14/553 bp, no gap) in the LSU; from *S. rodionovae* (ex-type 3C) by 8.6% (49/571 bp, five gaps) in the ITS and 3.7% (22/591 bp, no gap) in the LSU; from *S. circinatum* (ex-type CBS 654.89) by 6.8% (49/721 bp, five gaps) in the ITS and 5.4% (67/1,234 bp, five gaps) in the LSU; from *S. terrigenum* (ex-type KNUF-23-236) by 3.9% (23/580 bp, one gap) in the ITS and 3.3% (19/561 bp, four gaps) in the LSU; and from *S. tongrenense* (ex-type CGMCC 3.28994) by 6.6% (36/544 bp, six gaps) in the ITS and 2.4% (14/575 bp, one gap) in the LSU.

This species demonstrates notable inhibitory effects against various wood-decay fungi, revealing promising biocontrol potential (Klingström and Beyer 1965; Klingstrom and Johansson 1973; Cease et al. 1989). Furthermore, it produces diverse, unique metabolites (such as sorbicillinoid analogs) that exhibit significant inhibitory activity against cancer cells and *Aspergillus niger*, indicating considerable medicinal potential (El-Elimat et al. 2015).

#### 2.﻿ Scytalidium assmuthi

Taxon classificationAnimaliaHelotialesChaetomiaceae

G. Mane, R. Avchar, R. Morey & Rohit Sharma, Fungal Diversity 130: 86 (2024)

505F241A-5257-5887-BDB5-B5B1217BEA9E

##### Description and illustration.

Manawasinghe et al. (2024).

##### Notes.

*Scytalidium assmuthi* was introduced to accommodate an isolate obtained from the gut of the termite *Odontotermes assmuthi* feeding on wood logs from the northern Western Ghat (India) (Manawasinghe et al. 2024). *Scytalidium assmuthi* is phylogenetically closely related to *S. album*, *S. aurantiacum*, *S. rodionovae*, *S. circinatum*, *S. terrigenum*, and *S. tongrenense* (Figs 1, 2). The distinctions between *S. assmuthi* and *S. album* are provided in the notes for *S. album*. *Scytalidium assmuthi* is distinguished from *S. aurantiacum*, *S. rodionovae*, *S. circinatum*, *S. terrigenum*, and *S. tongrenense* by its unknown sexual morph and production of only chlamydospore-like cells (Klingström and Beyer 1965; Sigler and Wang 1990; Pavlov et al. 2018; Manawasinghe et al. 2024; Jeong et al. 2025). Furthermore, based on a pairwise comparison of ITS, *S. assmuthi* (ex-type PYCC 9837) differs from *S. aurantiacum* (ex-type CBS 374.65) by 8.2% (37/453 bp, 37 gaps) in the ITS and 9.9% (87/879 bp, 62 gaps) in the LSU; from *S. rodionovae* (ex-type 3C) by 3.7% (17/461 bp, four gaps) in the ITS and 0.5% (4/830 bp, one gap) in the LSU; from *S. circinatum* (ex-type CBS 654.89) by 3.3% (15/459 bp, two gaps) in the ITS and 0.9% (5/573 bp, one gap) in the LSU; from *S. terrigenum* (ex-type KNUF-23-236) by 8% (37/461 bp, five gaps) in the ITS and 2.9% (24/831 bp, two gaps) in the LSU; from *S. tongrenense* (ex-type CGMCC 3.28994) by 8.9% (40/447 bp, eight gaps) in the ITS and 4% (33/830 bp, two gaps) in the LSU. No additional studies are currently available regarding this species.

#### 3.﻿ Scytalidium aurantiacum

Taxon classificationAnimaliaHelotialesChaetomiaceae

L. Beyer & Klingström, Svensk bot. Tidskr. 59: 35 (1965)

D96D5526-A55E-5C02-A7DE-CE5F4394FC6E

##### Description and illustration.

Klingström and Beyer (1965).

##### Notes.

*Scytalidium aurantiacum* was established to accommodate strain FF21 isolated from pine pulpwood and strain FF29 obtained from birch (*Betula*) pulpwood (Klingström and Beyer 1965). *Scytalidium aurantiacum* is phylogenetically closely related to *S. album*, *S. assmuthi*, *S. rodionovae*, *S. circinatum*, *S. terrigenum*, and *S. tongrenense* (Figs 1, 2). The distinctions between *S. aurantiacum* and *S. album*/*S. assmuthi* are provided in the notes for *S. album* and *S. assmuthi*, respectively. *Scytalidium aurantiacum* is distinguished from *S. rodionovae*, *S. terrigenum*, and *S. tongrenense* by its unknown sexual morph and hyaline arthroconidia (Klingström and Beyer 1965; Pavlov et al. 2018; Jeong et al. 2025). *Scytalidium aurantiacum* differs from *S. circinatum* by its bacilliformia arthroconidia, globose, or ellipsoidal chlamydospore-like cells (Klingström and Beyer 1965; Sigler and Wang 1990). Additionally, based on a pairwise comparison of ITS and LSU, *S. aurantiacum* (ex-type CBS 374.65) differs from *S. rodionovae* (ex-type 3C) by 9% (49/531 bp, five gaps) in the ITS and 9.8% (88/896 bp, 61 gaps) in the LSU; from *S. circinatum* (ex-type CBS 654.89) by 8.9% (47/530 bp, five gaps) in the ITS and 3.2% (19/590 bp, no gap) in the LSU; from *S. terrigenum* (ex-type KNUF-23-236) by 2.5% (13/528 bp, one gap) in the ITS and 8.4% (822/897 bp, 62 gaps) in the LSU; from *S. tongrenense* (ex-type CGMCC 3.28994) by 6.3% (33/522 bp, six gaps) in the ITS and 8.8% (79/896 bp, 79 gaps) in the LSU.

This species not only causes blue stain in wood but also exhibits strong antagonistic effects against various wood-decaying fungi, particularly remaining effective at low temperatures (10 °C) (Klingström and Beyer 1965; Strunz et al. 1972; Cease et al. 1989). Furthermore, the antibiotic metabolite scytalidin produced by *S. aurantiacum* has demonstrated antifungal activity, making it a candidate for antifungal agent development (Ayer et al. 1993). Dirginčiutė-Volodkienė and Pečiulytė (2011) demonstrated that *S. aurantiacum* exhibits significant tolerance to copper pollution, indicating its potential as a bioindicator for copper contamination and a candidate species for bioremediation applications.

#### 4.﻿ Scytalidium auriculariicola

Taxon classificationAnimaliaHelotialesChaetomiaceae

W.H. Peng, Z.R. Yang bis & Qun Sun, FEMS Microbio Lett. 359(1): 75 (2014)

2ECA8771-AFF8-55BF-B76D-72729EA5DAA0

##### Description and illustration.

Peng et al. (2014).

##### Notes.

*Scytalidium auriculariicola* is phylogenetically closely related to *S. lignicola* and *S. philadelphianum* (Figs 1, 2). *Scytalidium auriculariicola* differs from *S. lignicola* by its larger arthroconidia (5–19 × 2–3 μm in *S. auriculariicola vs.* 5–8 × 2 µm in *S. lignicola*) and bigger chlamydospore-like cells (7–11.5 × 5–7.5 μm in *S. auriculariicola* vs. 7 µm in *S. lignicola*) (Pesante 1957; Peng et al. 2014). *Scytalidium auriculariicola* differs from *S. philadelphianum* in that its chlamydospore-like cells are barrel-shaped to oblong, 7–11.5 × 5–7.5 μm, while those of *S. philadelphianum* are globose to ellipsoid, 4–6 μm (Pesante 1957; Crous et al. 2022). Furthermore, based on a pairwise comparison of ITS, *S. auriculariicola* (ex-type YBI-3) differs from *S. lignicola* (ex-type UAMH 1502) by 10.1% (62/612 bp, 37 gaps) and from *S. philadelphianum* (ex-type CPC 40793) by 10.4% (64/612 bp, 36 gaps).

*Scytalidium auriculariicola* is the pathogen causing slippery scar in *Auricularia polytricha*, posing a significant threat to its cultivation (Peng et al. 2014). Chen et al. (2019) reported the mitochondrial genome characteristics of this species, which contributed to the detection and control of this pathogen. Furthermore, no other studies on this species have been reported.

#### 5.﻿ Scytalidium chlamydosporum

Taxon classificationAnimaliaHelotialesChaetomiaceae

S.Q. Tong & Zhi.Y. Zhang, sp. nov.

0BB5E09C-821C-5D9D-BB63-B623A6BBF496

859963

Fig. 3

##### Type.

CHINA • Guizhou, Guiyang, Xiuwen County, Liuguang Town, 26.99°N, 116.44°E, soil, 5 July 2022, Shuo-Qiu Tong (holotype HMAS 354096, dried culture; ex-type CGMCC 3.28993, *ibid*., SQT10)

##### Etymology.

Refers to the species that only produces chlamydospore-like cells.

##### Description.

***Culture characteristics*** (14 days at 25 °C): Colonies on PDA attaining 80–82 mm diam., flat, cottony, margin entire, gray-yellow (4A2) to pale gray (30B2). Reverse gray-yellow (4A2) to pale gray (30B2). Colonies on SNA fast-growing, more than 90 mm, flat, tomentose, pale gray (30B2). Reverse pale gray (30B2).

**Figure 3.**
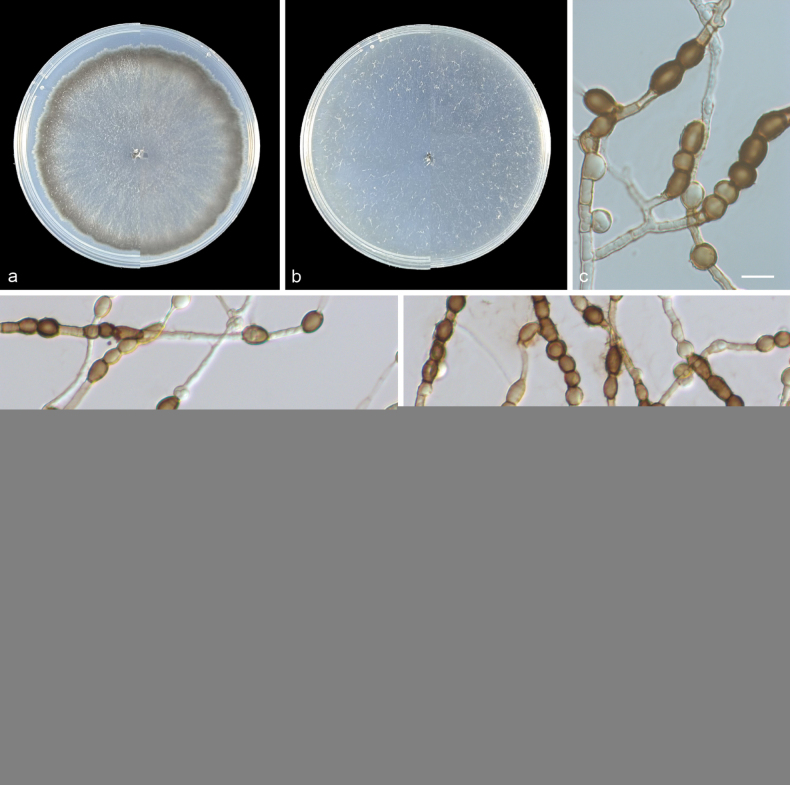
*Scytalidium chlamydosporum* (from holotype HMAS 354096) a, b upper and reverse views of cultures on PDA and SNA after 14 d at 25 °C c–e chlamydospore-like cells. Scale bars: 10 μm (c); 20 μm (d–e).

***Mycelium*** consisting of hyaline, smooth-walled, branched, septate, 1–3 μm diam hyphae. ***Chlamydospore-like*** cells yellow-brown to dark brown, oblong to globose, ellipsoidal, 0–1-septate, thick-walled, smooth, solitary, catenate, 6–11.5 × 6–8.5 µm (avg. 8.5 × 7.1 μm, n = 30). ***Conidia*** and ***Arthroconidia*** unknown. ***Sexual morph*** unknown.

##### Geographical distribution.

Guizhou Province, China.

##### Additional material examined.

CHINA • Guizhou, Guiyang, Huaxi District, Shiban Town, 26.46°N, 106.65°E, soil, 8 July 2022, Shuo-Qiu Tong, SQT11, ibid. SQT12.

##### GenBank numbers.

SQT10–SQT12, ITS: PV890025–PV890027, LSU: PV890032–PV890034.

##### Notes.

Phylogenetic analysis showed that three new isolates (SQT10–SQT12) clustered in a single subclade with high supported value (100/1) and were closely related to *S. cuboideum* and *S. sphaerosporum* (Figs 1, 2). Morphologically, *S. chlamydosporum* differs from *S. cuboideum* by its absent arthroconidia and production of chlamydospore-like cells (Kang et al. 2010). *Scytalidium chlamydosporum* differs from *S. sphaerosporum* by its absent sexual morph and production of chlamydospore-like cells (Kang et al. 2010). Furthermore, based on a pairwise comparison of ITS, *S. chlamydosporum* (ex-type CGMCC 3.28993) differs from *S. cuboideum* (ex-isotype UAMH 676) by 10.9% (60/549 bp, 21 gaps) and from *S. sphaerosporum* (ex-type ATCC 34392) by 12.4% (64/513 bp, 28 gaps). *Scytalidium chlamydosporum* was isolated from soil in a pepper cultivation area and is likely to have a saprophytic lifestyle.

#### 6.﻿ Scytalidium circinatum

Taxon classificationAnimaliaHelotialesChaetomiaceae

Sigler & C.J.K. Wang, Mycologia 82: 399 (1990)

757D69B1-507A-56DB-AE02-3A519FB7A259

##### Description and illustration.

Sigler and Wang (1990).

##### Notes.

*Scytalidium circinatum* was initially isolated and established by Sigler and Wang (1990) from preservative-treated utility poles. *Scytalidium circinatum* is phylogenetically closely related to *S. album*, *S. assmuthi*, *S. aurantiacum*, *S. rodionovae*, *S. terrigenum*, and *S. tongrenense* (Figs 1, 2). The distinctions between *S. circinatum* and *S. album*, *S. assmuthi*, *S. aurantiacum*, *S. rodionovae*, *S. terrigenum*, and *S. tongrenense* are detailed in the respective notes for *S. album*, *S. assmuthi*, *S. aurantiacum*, and *S. rodionovae*. *Scytalidium circinatum* is distinguished from *S. terrigenum* and *S. tongrenense* by the shape, size, and aseptate of its chlamydospore-like cells (aseptate, globose, lobed, or irregularly shaped, 4–9 × 3–9 μm in *S. circinatum* vs. septate, oval-shaped, 5.4–13 × 4.2–8.1 μm in *S. terrigenum*; and septate, oblong to globose, subcylindrical, guttulate, or irregular, measuring 5–12.5 × 4–6 μm in *S. tongrenense*) (Jeong et al. 2025). Additionally, based on a pairwise comparison of ITS and LSU, *S. circinatum* (ex-type CBS 654.89) differs from *S. terrigenum* (ex-type KNUF-23-236) by 7.2% (42/583 bp, six gaps) in the ITS and 4% (24/597 bp, two gaps) in the LSU; from *S. tongrenense* (ex-type CGMCC 3.28994) by 9.1% (50/547 bp, 11 gaps) in the ITS and 3.6% (22/612 bp, one gap) in the LSU.

This species exhibits capabilities for wood decay and resistance to chemical preservatives. It is widely distributed, having been reported in diverse environments such as underground mines in Minnesota, USA, urban trees in Singapore, and tobacco fields in Bijie, Guizhou, China (Held et al. 2020; Wang et al. 2022; Hong et al. 2023).

#### 7.﻿ Scytalidium cuboideum

Taxon classificationAnimaliaHelotialesChaetomiaceae

(Sacc. & Ellis) Sigler & Kang, Mycologia 102(5): 1179 (2010)

96AA98A3-2005-5929-8F46-03393ABC093C

Geotrichum microsporum Smith, Trans. Br. Mycol. Soc. 45: 388. 1962.

##### Basionym.

*Oospora cuboidea* Sacc. & Ellis, Michelia 2(8): 576. 1882.

##### Synonym.

*Arthrographis cuboidea* (Sacc. & Ellis) Sigler, Mycotaxon 4: 363. 1976.

Other synonyms and a detailed description are provided in Sigler and Carmichael (1976).

##### Description and illustration.

Kang et al. (2010).

##### Notes.

*Scytalidium cuboideum* is phylogenetically closely related to *S. sphaerosporum* and *S. chlamydosporum* (Figs 1, 2). The distinctions between *S. cuboideum* and *S. chlamydosporum* are provided in the notes for *S. chlamydosporum*. Morphologically, *S. cuboideum* differs from *S. sphaerosporum* by its unknown sexual morph (Kang et al. 2010). Furthermore, based on a pairwise comparison of ITS, *S. cuboideum* (Isotype UAMH 676) differs from *S. sphaerosporum* (ex-type ATCC 34392) by 13.2% (73/554 bp, 23 gaps) in the ITS.

*Scytalidium cuboideum* is a soft rot fungus capable of causing pink or blue spalting in wood, commonly found on both hardwoods and softwoods such as oak (*Quercus* spp.) (Robinson et al. 2007). It has a global distribution and has been isolated from various regions, including the United States, South Africa, Japan, and Bhutan (Robinson et al. 2007). *Scytalidium cuboideum* produces a red pigment known as draconin red, which exhibits excellent lightfastness, UV resistance, and colorfastness, showing strong potential for applications in textiles, wood coatings, and paper dyeing (Robinson et al. 2014; Gutierrez et al. 2019; Hinsch et al. 2022; Diplock et al. 2023). Notably, although not a common pathogen, it has been isolated from respiratory samples (such as bronchial wash and lung tissue), can grow at 37 °C, and is susceptible to several antifungal agents (e.g., posaconazole, voriconazole) but resistant to echinocandins and terbinafine, indicating potential pathogenicity (Giraldo et al. 2013; Sy-Cordero et al. 2015).

#### 8.﻿ Scytalidium ganodermophthorum

Taxon classificationAnimaliaHelotialesChaetomiaceae

Kang, Sigler, Lee & Yun, Mycologia 102(5): 1179 (2010)

A7CBDBF8-B616-5E5E-91C9-0E40481DC7C1

Xylogone ganodermophthora Kang, Sigler, Lee & Yun, Mycologia 102(5): 1179. 2010.

##### Synonym.

*Scytalidium parasiticum* Yit K. Goh, Goh, Y.K. Goh & K.J. Goh, Mycobiology 43(2): 112. 2015.

##### Description and illustration.

Kang et al. (2010).

##### Notes.

*Scytalidium ganodermophthorum* is phylogenetically closely related to *S. synnematicum* (Figs 1, 2). Morphologically, *S. ganodermophthorum* differs from *S. synnematicum* by its production of a sexual morph, arthroconidia, and not producing conidiomata or synnemata (Kang et al. 2010; Crous et al. 2023). Furthermore, based on a pairwise comparison of ITS, *S. ganodermophthorum* (ex-type UAMH 10320) differs from *S. synnematicum* (ex-type CCMB207/13) by 9.6% (50/520 bp, 16 gaps) in the ITS. Goh et al. (2015) established *S. parasiticum* based on morphological characteristics and phylogenetic analyses. However, in this study, *S. parasiticum* (ex-type AAX0113) and *S. ganodermophthorum* (UAMH 10320, H123, and TPML 97003) clustered in a single subclade with high supported value (100/1) (Figs 1, 2). In a comparison of ITS, *S. parasiticum* (AAX0113) exhibited 99.8% (515/516 bp, no gap) similarity to *S. ganodermophthorum* (ex-type UAMH 10320). Moreover, the morphological characteristics between *S. parasiticum* and *S. ganodermophthorum* are minor; therefore, we treat *S. parasiticum* as a synonym of *S. ganodermophthorum*.

*Scytalidium ganodermophthorum* was first discovered in Korea as a pathogenic fungus causing yellow rot in cultivated *Ganoderma lucidum* (Kang et al. 2010), on which it behaves as an obligate or facultative parasite. While *S. ganodermophthorum* is a pathogen of *G. lucidum*, its synonym *S. parasiticum* has been shown to exhibit antagonistic effects against another *Ganoderma* species, the oil palm pathogen *G. boninense*, demonstrating its potential as a biocontrol agent (Goh et al. 2016).

#### 9.﻿ Scytalidium guizhouense

Taxon classificationAnimaliaHelotialesChaetomiaceae

S.Q. Tong & Zhi.Y. Zhang sp. nov.

40C9538C-24F9-5501-BBC7-EB01A3F6CFD4

859964

Fig. 4

##### Type.

CHINA • Guizhou, Guiyang, Huaxi District, Shiban Town, 26.46°N, 106.65°E, soil, 8 July 2022, Shuo-Qiu Tong (holotype HMAS 354097, dried culture; ex-type CGMCC 3.28992, *ibid*., SQT08 and SQT09).

##### Etymology.

The epithet refers to the type location.

##### Description.

***Culture characteristics*** (14 days at 25 °C): Colony on PDA attaining 48–55 mm diam., flat, margin lobate edge, ash gray (1B2) to dull green (30E3). Reverse ash gray (1B2) to jade green (27E3). Colony on SNA attaining 20–26 mm diam., flat, margin radially striate with lobate edge, white (1A1). Reverse white (1A1).

***Mycelium*** consisting of hyaline, smooth-walled, branched, septate, 1–2.5 μm diam hyphae. ***Conidiophores*** erect, hyaline, smooth, solitary, terminal, lateral, subcylindrical, straight to flexuous, unbranched, 0–2-septate, reduced to conidiogenous cells, 12.5–43 × 1–3 μm. ***Conidia*** solitary, catenate, hyaline, smooth-walled, oblong or ellipsoidal to globose, subcylindrical to cylindrical, not constricted at septa, segments 6.5–12 × 5–7 μm (avg. 8.9 × 5.9 μm, n = 30). ***Chlamydospore-like*** cells dark brown, oblong or ellipsoidal to subglobose, ellipsoidal, 0–1-septate, thick-walled, smooth, solitary or catenate, 7.5–13 × 4.5–6.5 μm (avg. 10.5 × 5.4 μm, n = 30). Fertile hyphae borne laterally on simple conidiophores, fragmenting into arthroconidia. ***Arthroconidia*** hyaline to brown, smooth, cuboidal to oblong or cylindrical, 3–7.5 × 2–3 µm (avg. 5.3 × 2.7 μm, n = 30). ***Sexual morph*** unknown.

**Figure 4.**
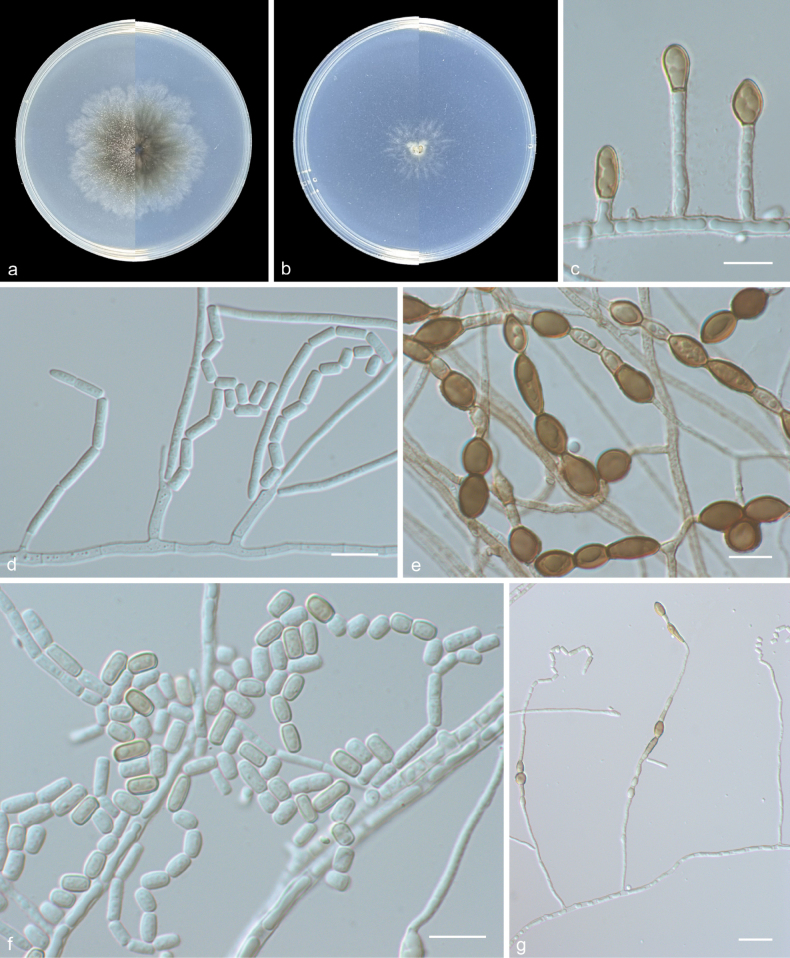
*Scytalidium guizhouense* (from holotype HMAS 354097) a, b upper and reverse views of cultures on PDA and SNA after 14 d at 25 °C c conidia and conidiophores d, f arthroconidia e chlamydospore-like cells g arthroconidia and chlamydospore-like cells. Scale bars: 10 μm (c–f); 20 μm (g).

##### Geographical distribution.

Guizhou Province, China.

##### GenBank numbers.

SQT08–SQT09, ITS: PV890023–PV890024, LSU: PV890030–PV890031.

##### Notes.

Phylogenetic analysis showed that two new isolates (CGMCC 3.28992 and SQT09) clustered in a single subclade with strong support (100/1) (Figs 1, 2). Morphologically, *S. guizhouense* differs from other species in *Scytalidium* by its production of conidia, arthroconidia, and chlamydospore-like cells, and an unknown sexual morph (see notes of key). *Scytalidium guizhouense* was isolated from soil in a pepper cultivation area and is likely to have a saprophytic lifestyle.

#### 10.﻿ Scytalidium lignicola

Taxon classificationAnimaliaHelotialesChaetomiaceae

Pesante, Ann. Sperim. Agrar. 11 (suppl.): 265 (1957)

C0A12407-A4E8-5AAA-8366-BFF88FF9654D

859964

##### Description and illustration.

Pesante (1957)

##### Notes.

*Scytalidium lignicola* is phylogenetically closely related to *S. auriculariicola* and *S. philadelphianum* (Figs 1, 2). The distinctions between *S. lignicola* and *S. auriculariicola* are provided in the notes for *S. auriculariicola*. Morphologically, *S. lignicola* differs from *S. philadelphianum* by not producing conidia and by having chlamydospore-like cells swollen up to 7 µm wide (Pesante 1957; Crous et al. 2022). Furthermore, based on a pairwise comparison of ITS, *S. lignicola* (ex-type UAMH 1502) differs from *S. philadelphianum* (ex-type CPC 40793) in 5.8% (34/580 bp, 14 gaps) in the ITS. *Scytalidium lignicola* acts as a saprotrophic opportunist in wood, soil, and compost but can shift to a pathogenic mode of life, causing infections in humans (Dickinson et al. 1983; De Gannes et al. 2013). Notably, as a soil-borne pathogen, *Scytalidium lignicola* often causes cassava black root rot, which is difficult to control and results in significant losses in cassava production (Silva et al. 2013).

#### 11.﻿ Scytalidium philadelphianum

Taxon classificationAnimaliaHelotialesChaetomiaceae

Crous & Jurjević, Fungal Syst. Evol. 10: 84 (2022)

BD6FE758-5BF1-5B87-86D8-EE771C7A6782

859964

##### Description and illustration.

Crous et al. (2022).

##### Notes.

*Scytalidium philadelphianum* was initially isolated from compressed air in a factory located in Philadelphia, Pennsylvania, USA (Crous et al. 2022). *Scytalidium philadelphianum* is phylogenetically closely related to *S. auriculariicola* and *S. lignicola* (Figs 1, 2). The distinctions between *S. philadelphianum* and *S. auriculariicola* are provided in the notes for *S. auriculariicola*, while the distinctions between *S. philadelphianum* and *S. lignicola* are provided in the notes for *S. lignicola*. Notably, Ringhofer et al. (2025) obtained the strain UTHSCSA DI24-300 from urine and intervertebral disc aspirate cultures of a 3-year-old male Belgian Malinois presenting with progressive hindlimb weakness. The isolate was identified as *S. philadelphianum* based on 100% similarity of its ITS with that of ex-type strain CPC 40793.

#### 12.﻿ Scytalidium rodionovae

Taxon classificationAnimaliaHelotialesChaetomiaceae

S.Q. Tong & Zhi.Y. Zhang sp. nov.

9BAAD4B8-D64D-525E-AB52-3C9435337257

860661

##### Type.

RUSSIA • Moscow, isolated from a rotting rope in the 1970s, living culture 3C.

##### Etymology.

It was initially isolated by Rodionova et al.

##### Description and illustration.

Pavlov et al. (2018).

##### Notes.

*Scytalidium rodionovae* is phylogenetically closely related to *S. album*, *S. assmuthi*, *S. aurantiacum*, *S. circinatum*, *S. terrigenum*, and *S. tongrenense* (Figs 1, 2). The distinctions between *S. rodionovae* and *S. album*, *S. assmuthi*, and *S. aurantiacum* are detailed in the respective notes for *S. album*, *S. assmuthi*, and *S. aurantiacum*. *Scytalidium rodionovae* is distinguished from *S. circinatum*, *S. terrigenum*, and *S. tongrenense* by its known sexual morph, hyaline, ovoid, and septate chlamydospore-like cells (Sigler and Wang 1990; Jeong et al. 2025). Additionally, based on a pairwise comparison of ITS and LSU, *S. rodionovae* (ex-type 3C) differs from *S. circinatum* (ex-type CBS 654.89) by 4% (23/571 bp, four gaps) in the ITS and 1.1% (7/628 bp, no gap) in the LSU; from *S. terrigenum* (ex-type KNUF-23-236) by 7.8% (44/559 bp, six gaps) in the ITS and 3.2% (42/1299 bp, two gaps) in the LSU; from *S. tongrenense* (ex-type CGMCC 3.28994) by 9.7% (53/548 bp, 11 gaps) in the ITS and 3.7% (34/909 bp, one gap) in the LSU.

Rodionova et al. (1974) identified strain 3C as *Geotrichum candidum* based only on morphological characters. Pavlov et al. (2018) integrated morphological characteristics and phylogenetic analyses based on molecular data derived from 3C to reclassify *G. candidum* into *Scytalidium*, naming it *S. candidum*. However, they overlooked the fact that 3C does not belong to *G. candidum*. Here, we propose the new species *Scytalidium rodionovae* to accommodate the species for which 3C is the ex-type. To date, the genome sequencing of strain 3C has been completed (Polev et al. 2014). Studies have shown that this strain secretes a variety of enzymes capable of efficiently degrading cellulose, exhibits broad pH adaptability, and holds potential application value in lignocellulosic biomass conversion and green industrial processes (Lapin et al. 2002; Borisova et al. 2015).

#### 13.﻿ Scytalidium sphaerosporum

Taxon classificationAnimaliaHelotialesChaetomiaceae

Sigler & Kang, Mycologia 102(5): 1179 (2010)

FEACC8B1-2187-55B1-8094-2C5D5B4995FA

##### Description and illustration.

Kang et al. (2010).

##### Notes.

*Scytalidium sphaerosporum* was initially isolated from wood chips of pine (*Pinus sylvestris*) in Sweden (Kang et al. 2010). *Scytalidium sphaerosporum* is phylogenetically closely related to *S. chlamydosporum* and *S. cuboideum* (Figs 1, 2). However, the distinctions between *S. sphaerosporum* and *S. chlamydosporum* are provided in the notes for *S. chlamydosporum*, while the distinctions between *S. sphaerosporum* and *S. cuboideum* are provided in the notes for *S. cuboideum*.

*Scytalidium sphaerosporum* is commonly found in wood chips of both coniferous and broad-leaved trees, as well as in preservative-treated timber, and has no pathogenic relationship with *Ganoderma* (Kang et al. 2010; Goh et al. 2015).

#### 14.﻿ Scytalidium synnematicum

Taxon classificationAnimaliaHelotialesChaetomiaceae

G.G. Barreto & Gusmão, Persoonia 50: 287 (2023)

13B606E0-9317-58F2-8670-26FD4B360EBE

##### Description and illustration.

Crous et al. (2023).

##### Notes.

*Scytalidium synnematicum* was isolated by Crous et al. (2023) as a saprobe from dead twigs of an unidentified plant collected in Amazonas, Brazil. *Scytalidium synnematicum* is phylogenetically closely related to *S. ganodermophthorum* (Figs 1, 2). However, the distinctions between *S. synnematicum* and *S. ganodermophthorum* are provided in the notes for *S. ganodermophthorum*. To date, *S. synnematicum* is the only species within the genus *Scytalidium* that produces synnemata (Crous et al. 2023).

#### 15.﻿ Scytalidium terrigenum

Taxon classificationAnimaliaHelotialesChaetomiaceae

Y.S. Jeong, S.Yeol Lee & H.Y. Jung, Mycobiology 53(3): 297 (2025)

EF897547-6204-5AFB-A683-9A0A40974F3F

##### Description and illustration.

Jeong et al. (2025).

##### Notes.

*Scytalidium terrigenum* was isolated and named by Jeong et al. (2025) from a soil sample collected in Chungcheongnam-do, South Korea. *Scytalidium terrigenum* is phylogenetically closely related to *S. album*, *S. assmuthi*, *S. aurantiacum*, *S. rodionovae*, *S. circinatum*, and *S. tongrenense* (Figs 1, 2). However, the distinctions between *S. terrigenum* and *S. album*, *S. assmuthi*, *S. aurantiacum*, *S. rodionovae*, and *S. circinatum* are provided in their respective notes. Additionally, *S. terrigenum* differs from *S. tongrenense* by its production of hyaline to brown arthroconidia and oval chlamydospore-like cells (Jeong et al. 2025). Furthermore, based on a pairwise comparison of ITS and LSU, *S. terrigenum* (ex-type KNUF-23-236) differs from *S. tongrenense* (ex-type CGMCC 3.28994) by 5.5% (30/544 bp, seven gaps) in the ITS and 2.4% (22/895 bp, five gaps) in the LSU. Notably, *S. terrigenum* is capable of growing in acidic (pH 4) and low-temperature (10 °C) environments (Jeong et al. 2025).

#### 15.﻿ Scytalidium tongrenense

Taxon classificationAnimaliaHelotialesChaetomiaceae

S.Q. Tong & Zhi.Y. Zhang sp. nov.

EA051DF1-CD36-5AD4-9E0F-8C266A1977C0

859965

Fig. 5

##### Type.

CHINA • Guizhou, Tongren, Yanhe County, Qiaojia Town, 28.28°N, 108.44°E, soil, 5 July 2023, Shuo-Qiu Tong (holotype HMAS 354098, dried culture; ex-type CGMCC 3.28994, *ibid*., SQT13)

##### Etymology.

The epithet refers to the type location.

##### Description.

***Culture characteristics*** (14 days at 25 °C): Colonies on PDA attaining 17–34 mm diam., flat, margin fimbriate, floral white (1A2) to white (1A1). Reverse floral white (1A2) to white (1A1). Colonies on SNA attaining 66–68 mm diam., flat, margin entire, pale gray (30B2). Reverse pale gray (30B2).

**Figure 5.**
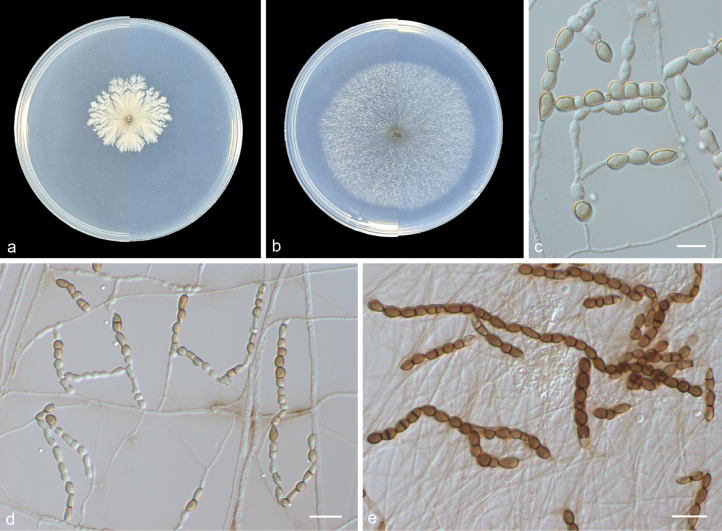
*Scytalidium tongrenense* (from holotype HMAS 354098) a, b upper and reverse views of cultures on PDA and SNA after 14 d at 25 °C c–e chlamydospore-like cells. Scale bars: 10 μm (c); 20 μm (d, e).

***Chlamydospore-like*** cells yellow-brown to dark brown, oblong to globose, ellipsoidal, subcylindrical, guttulate, or irregular, 0–1-septate, thick-walled, smooth, catenate, 5.5–12 × 4.5–6 µm (avg. 8.1 × 5.2 μm, n = 30). ***Arthroconidia*** unknown. ***Sexual morph*** unknown.

##### Geographical distribution.

Guizhou Province, China.

##### Additional material examined.

CHINA • Guizhou, Guiyang, Huaxi District, Shiban Town, 26.46°N, 106.65°E, soil, 8 July 2022, Shuo-Qiu Tong, SQT14.

##### GenBank numbers.

SQT13–SQT14, ITS: PV890028–PV890029, LSU: PV890035–PV890036.

##### Notes.

Phylogenetic analysis showed that two new isolates (SQT13 and SQT14) clustered in a single subclade with high supported value (100/1) and are sister to *S. album*, *S. assmuthi*, *S. aurantiacum*, *S. rodionovae*, *S. circinatum*, and *S. terrigenum* (Figs 1, 2). However, the distinctions between *S. tongrenense* and *S. album*, *S. assmuthi*, *S. aurantiacum*, *S. rodionovae*, *S. circinatum*, and *S. terrigenum* are provided in their respective notes. *Scytalidium tongrenense* was isolated from soil in a pepper cultivation area and is likely to have a saprophytic lifestyle.

#### ﻿*Neocosmospora* E.F. Sm., Bull. U.S.D.A. 17: 45. 1899.

##### 15.﻿ Scytalidium xizangensis

Taxon classificationAnimaliaHelotialesChaetomiaceae

(Y.H. Geng & T.Y. Zhang) S.Q. Tong & Zhi.Y. Zhang comb. nov.

E1D67414-F813-580A-89B1-BEFAD63CF91B

860664

###### Basionym.

*Scytalidium tuberculatum* Y.H. Geng & T.Y. Zhang, Mycosystema 35(11): 1313. 2016.

###### Description and illustration.

Geng et al. (2016).

###### Notes.

*Scytalidium tuberculatum* was introduced by Geng et al. (2016). In this study, the ex-type strain (H1195) of *S. tuberculatum* nested in the genus *Neocosmospora* (Fig. 1). However, the name *Neocosmospora tuberculata* is already occupied (Lee et al. 2025). Therefore, we transferred *S. tuberculatum* to the genus *Neocosmospora* as *Neocosmospora xizangensis*.

### ﻿*Mycothermus* D.O. Natvig, J.W. Taylor, A. Tsang, M.I. Hutch. & A.J. Powell ex X. Wei Wang, Houbraken & D.O. Natvig, Stud. Mycol. 93: 107. 2018.

#### ﻿ Cladosporium thermophilus

Taxon classificationAnimaliaHelotialesChaetomiaceae

(Cooney & R. Emers.) X. Wei Wang, Houbraken & D.O. Natvig, Stud. Mycol. 93: 107. 2018.

E4676643-AADF-56E7-A598-A3DAEAFB6003

Mycothermus thermophilus (Cooney & R. Emers.) D.O. Natvig et al., Mycologia 107: 321. 2015, nom. inval.
Humicola insolens Cooney & R. Emers., Thermophilic Fungi: 72. 1964.
Humicola grisea var. thermoides Cooney & R. Emers., Thermophilic Fungi: 72. 1964.
Scytalidium indonesiacum Hedger, Samson & Basuki, Trans. Brit. Mycol. Soc. 78(2): 365. 1982.

##### Basionym.

*Torula thermophila* Cooney & R. Emers., Thermophilic Fungi: 92. 1964.

##### Synonyms.

*Scytalidium thermophilum* (Cooney & R. Emers.) Austwick, New Zealand J. Agric. Res. 19: 29. 1976.

##### Description and illustration.

Wang et al. (2019).

##### Notes.

Wang et al. (2019) validated *Mycothermus thermophilus*. In this study, the ex-type strain (CBS 259.81) of *S. indonesiacum* nested in the genus *Mycothermus* (Fig. 1). Although the clade containing *S. indonesiacum* (CBS 259.81) and *M. thermophilus* (CBS 625.91) received low statistical support (80/-), they exhibit minimal morphological and sequence divergence. The ITS and LSU sequence similarities between *S. indonesiacum* (CBS 259.81) and *M. thermophilus* (CBS 625.91) were 99% and 99.5%, respectively. Therefore, we synonymize *S. indonesiacum* under *M. thermophilus*.

### ﻿*Hypoxylon* Bull., Histoire des champignons de la France: 168. 1791.

#### ﻿ Cladosporium terminale

Taxon classificationAnimaliaHelotialesChaetomiaceae

(G.V. Rao & de Hoog) S.Q. Tong & Zhi.Y. Zhang comb. nov.

0B130982-5410-5FAB-8024-43EA7B5F43AC

860666

##### Synonyms.

*Scytalidium terminale* G.V. Rao & de Hoog, Persoonia 8(2): 203. 1975.

##### Description and illustration.

Rao and Hoog (1975).

##### Notes.

*Scytalidium terminale* was introduced by Rao and Hoog (1975). In this study, the ex-type strain (CBS 171.40) of *S. terminale* nested in the genus *Hypoxylon* (Fig. 1). Therefore, we transferred *S. terminale* to the genus S*cytalidium* as *H. terminale*.

#### ﻿*Monochaetia* (Sacc.) Allesch., Rabenhorst’s Kryptogamen-Flora, Pilze - Fungi Imperfecti Ed. 2, 1(7): 665. 1902.

##### ﻿ Cladosporium dimorphospora

Taxon classificationAnimaliaHelotialesChaetomiaceae

T. Yokoy., Trans. Brit. Mycol. Soc. 65(3): 500. 1975.

D6F44FD9-20B1-517F-800B-BEDB9F21673C

###### Synonyms.

*Scytalidium flavobrunneum* (J.H. Mill., Giddens & A.A. Foster) Sigler, Mycotaxon 4(2): 400. 1976.

###### Description and illustration.

Yokoyama (1975).

###### Notes.

*Monochaetia dimorphospora* was introduced by Yokoyama (1975). In this study, the ex-type strain (CBS 244.59) of *S. flavobrunneum* nested in the genus *Monochaetia* and is closely related to *M. dimorphospora* (ex-type NBRC 9980) (Fig. 1). Additionally, *S. flavobrunneum* and *M. dimorphospora* exhibit minimal morphological and sequence divergence. The ITS-LSU sequence similarities between *S. flavobrunneum* (CBS 244.59) and *M. dimorphospora* (NBRC 9980) were 99.9%. Therefore, we synonymize *S. flavobrunneum* under *M. dimorphospora*.

#### ﻿*Neodevriesia* Quaedvl. & Crous, Persoonia 33: 24. 2014.

##### ﻿ Cladosporium infestans

Taxon classificationAnimaliaHelotialesChaetomiaceae

(Iwatsu, Udagawa & Hatai) S.Q. Tong & Zhi.Y. Zhang comb. nov.

28EDB6DD-D8B5-502E-907B-469F23841937

860667

###### Basionym.

*Scytalidium infestans* Iwatsu, Udagawa & Hatai, Trans. Mycol. Soc. Japan 31(3): 389. 1990.

###### Synonyms.

*Neodevriesia cladophorae* M.M. Wang & W. Li, Mycologia 109(6): 967. 2018.

###### Description and illustration.

Iwatsu et al. (1990).

###### Notes.

*Scytalidium infestans* was introduced by Iwatsu et al. (1990). *Neodevriesia cladophorae* was introduced by Wang et al. (2017). In this study, the ex-type strain (CBS 161.91) of *S. infestans* nested in the genus *Neodevriesia* and was closely related to *N. cladophorae* (ex-type CGMCC 3.17901) (Fig. 1). Additionally, both *S. infestans* and *N. cladophorae* share similar conidia and chlamydospore-like cell morphology and dimensions (Iwatsu et al. 1990; Wang et al. 2017), along with highly conserved sequences. The ITS and LSU sequence similarities between *S. infestans* (CBS 161.91) and *N. cladophorae* (CGMCC 3.17901) were 99.6% and 100%, respectively. Since *S. infestans* was published prior to *N. cladophorae*, we transfer *S. infestans* to *Neodevriesia* as *Neodevriesia infestans*, with *N. cladophorae* being synonymized under it.

#### ﻿*Caliciopsis* Peck, Rep. (Annual) New York State Mus. Nat. Hist. 33: 32. 1880.

##### ﻿ Caliciopsis uredinicola

Taxon classificationAnimaliaHelotialesChaetomiaceae

(Kuhlman, J.W. Carmich. & T. Mill.) Zhi.Y. Zhang comb. nov.

EEE1184A-3BB1-5DEE-87B0-0BD0128706AF

860668

###### Basionym.

*Scytalidium uredinicola* Kuhlman, J.W. Carmich. & T. Mill., Mycologia 68(6): 1189. 1976.

###### Description and illustration.

Kuhlman et al. (1976).

###### Notes.

*Scytalidium uredinicola* was introduced by Kuhlman et al. (1976). In this study, the ex-type strain (CBS 578.75) of *S. uredinicola* nested in the genus *Caliciopsis* and was closely related to *C. pinea* (CBS 139.64) (Fig. 1). However, they can be distinguished by their low sequence similarities. The ITS sequence similarities between *S. uredinicola* (CBS 578.75) and *C. pinea* (CBS 139.64) were 97.1%. Therefore, we transfer *S. uredinicola* to *Caliciopsis* as *Caliciopsis uredinicola*.

### ﻿Excluded species

#### ﻿1. Scytalidium multiseptatum

Taxon classificationAnimaliaHelotialesChaetomiaceae

Hol.-Jech., Česká Mykol. 44(2): 101. 1990.

B3444F07-F7A2-59EC-97AE-E004C8B1EAF4

##### Description and illustration.

Holubová-Jechová (1990).

##### Notes.

In the phylogenetic tree (Fig. 1), *S. multiseptatum* (CBS 693.70 and CBS 241.68) formed a distinct clade within *Tricladiaceae*. Due to the lack of molecular sequence data from the ex-type strain (CBS 136.91) of *S. multiseptatum*, we excluded this species from *Scytalidium*, but further data are required to clarify its taxonomic status.

#### ﻿2. Scytalidium tibetense

Taxon classificationAnimaliaHelotialesChaetomiaceae

Y.H. Geng & T.Y. Zhang, Mycosystema 35(11): 1312. 2016.

F208F966-84FF-545C-B2C2-A436F6D0D4C2

##### Description and illustration.

Geng et al. (2016).

##### Notes.

In the phylogenetic tree (Fig. 1), *S. tibetense* (ex-type H1127) nested in the genus *Fusarium*. *Fusarium* is one of the most species-rich genera in *Sordariomycetes*, and its species identification requires a combination of multiple approaches. Therefore, we excluded *S. tibetense* from *Scytalidium*, but further studies are needed to determine its precise taxonomic status.

#### 3. Scytalidium japonicum

Taxon classificationAnimaliaHelotialesChaetomiaceae

Udagawa, K. Tominaga & Hamaoka, Mycotaxon 25(1): 281 (1986)

18468A4F-85CD-5D41-8657-47BF1F5D2C30

##### Description and illustration.

Udagawa et al. (1986).

##### Notes.

In the phylogenetic tree (Fig. 1), the two strains of *S. japonicum*, CBS 494.88 (ex-type) and CBS 125804, clearly separated into two distinct clades. The CBS 494.88 formed an independent subclade within *Dothideomycetes*, and further studies are required to clarify its taxonomic status. Additionally, the CBS 125804 was embedded within *Neoscytalidium* and showed a close relationship with CBS 145.78 (ex-type of *N. dimidiatum*), sharing 99.6% ITS-LSU sequence similarity. However, due to the lack of morphological characterization for CBS 125804, we refrain from assigning it to *N. dimidiatum* at this time.

#### 4. Scytalidium chinense

Taxon classificationAnimaliaHelotialesChaetomiaceae

Y.H. Geng & T.Y. Zhang, Mycosystema 35(11): 1311 (2016)

6AF77ACA-365E-57A7-914F-F975EF8FEF3E

##### Description and illustration.

Geng et al. (2016).

##### Notes.

In the phylogenetic tree (Fig. 1), the H1091 (ex-type of *Scytalidium chinense*) formed an independent subclade within *Dothideomycetes*, and further studies are required to clarify its taxonomic status.

### ﻿Uncertain species

During our compilation of literature and molecular data on *Scytalidium*, we identified several species for which no sequence data were available. Phylogenetic analyses revealed that multiple morphologically defined species originally classified in *Scytalidium* do not belong to this genus. Consequently, we designate these species as *incertae sedis*.

#### 1. Scytalidium fulvum

Taxon classificationAnimaliaHelotialesChaetomiaceae

Morgan-Jones & Gintis, Mycologia 76(2): 214. 1984.

FA53002B-F0C1-551A-8886-E2AE5608974C

##### Description and illustration.

Morgan-Jones et al. (1984).

#### 2. Scytalidium hepiali

Taxon classificationAnimaliaHelotialesChaetomiaceae

C. Lan Li, Acta Mycol. Sin. 7(1): 24. 1988.

6EFC49FB-80A7-51CE-97CC-C79DFA6FF612

##### Description and illustration.

Li and Sun (1988).

#### 3. Scytalidium melanoxylicola

Taxon classificationAnimaliaHelotialesChaetomiaceae

N. Awasthi, A. Dubey, S. Bhardwaj & A.N. Rai, Kavaka 55: 108. 2020.

66299F99-6DE6-5C87-B4AB-9C62E7391C56

##### Description and illustration.

Awasthi et al. (2020).

#### 4. Scytalidium nielamuense

Taxon classificationAnimaliaHelotialesChaetomiaceae

Y.M. Wu & T.Y. Zhang, Mycotaxon 114: 205. 2011.

CB5168B7-81B3-522D-ADCA-4F6730D44AFF

##### Description and illustration.

Wu and Zhang (2010).

#### 5. Scytalidium verruculosum

Taxon classificationAnimaliaHelotialesChaetomiaceae

Y.M. Wu & T.Y. Zhang, Mycotaxon 114: 207. 2011.

455F9772-9258-5CA5-88B7-51479478D6F9

##### Description and illustration.

Wu and Zhang (2010).

#### 6. Scytalidium xigazense

Taxon classificationAnimaliaHelotialesChaetomiaceae

Y.M. Wu & T.Y. Zhang, Mycotaxon 114: 209. 2011.

6B5AAB6F-9672-5879-BC7A-320A4CB60128

##### Description and illustration.

Wu and Zhang (2010).

## ﻿Discussion

To date, the taxonomic status of *Scytalidium* remains unresolved. Based on large-scale molecular data from *Leotiomycetes*, Ekanayaka et al. (2019) placed *Scytalidium* within *Helotiales* under *Hyaloscyphaceae* in their phylogenetic analysis. However, their sampling only included sequences from *S. vaccinii* (a synonym of *Hyaloscypha hepaticicola*) (CBS 652.89), while omitting the type species *S. lignicola*. In the same year, Johnston et al. (2019) focused on type species within *Leotiomycetes* and conducted a phylogenetic analysis using 15 concatenated genes, indicating that *Scytalidium* occupies a basal position within *Helotiales*, albeit with low resolution. Their data, however, were derived from strain IHIA52 (i.e., DSM 105466 in GenBank, identified as *S. lignicola*), yet the ITS sequence (MG815782) of this strain shows a 10.1% divergence (60/593, 28 gaps) compared to the ITS sequence of the type strain of *S. lignicola* (UAMH 1502; AY762623). Furthermore, after Ellis (1971) defined *Scytalidium* as fungi with darkly pigmented conidia, Sigler and Carmichael (1976) included several additional species based on morphological characteristics, though these species are not necessarily phylogenetically related. This has resulted in an overly broad concept of the genus, which urgently requires revision.

*Scytalidium* is characterized by two asexual morphs: one with dematiaceous intercalary conidia and another with hyaline, bacilliform arthroconidia (Pesante 1957; Ellis 1971). The arthroconidia are smooth, occasionally verrucose in age, mid or dark brown, cylindrical, oblong, doliform, or broadly ellipsoidal, and often 0-septate. Fission arthroconidia of a second type are hyaline or pale to mid-brown, smooth, cylindrical, 0-septate, and truncate at each end. Based on the original descriptions of 16 species within *Scytalidium* sensu stricto (Pesante 1957; Sigler and Wang 1990; Kang et al. 2010; Peng et al. 2014; Goh et al. 2015; Pavlov et al. 2018; Crous et al. 2022; Jeong et al. 2025), we propose that the characteristics of this genus are: ascomata cleistothecial, initially subhyaline, becoming dark brown at maturity with a wall of textura epidermoidea, without appendages, globose; asci subglobose to globose, quickly evanescent, four- or eight-spored; ascospores smooth, hyaline, subglobose to globose; conidia solitary or catenate, hyaline to dark brown; chlamydospore-like cells solitary or catenate, hyaline to dark brown, 0–1-septate, oblong to globose, subcylindrical, guttulate, or irregular; arthroconidia hyaline to light yellow, cuboidal to oblong or cylindrical.

Traditional fungal taxonomy has predominantly relied on morphological characteristics for species classification and description (Hawksworth 2001). However, phenotypic convergence frequently obscures phylogenetic relationships, rendering morphology-based identification unreliable for certain taxa (Bickford et al. 2007; Otálora et al. 2017). Integrated taxonomy, which uses multiple methods to identify and characterize fungal species, is currently being embraced by the scientific community (Zhang et al. 2024). At present, increasing numbers of studies combine morphological characteristics and molecular phylogenetic reconstruction for identifying fungal species. This approach effectively reduces the taxonomic misclassification of phenotypically similar but phylogenetically distinct lineages. For instance, relying solely on morphological characteristics makes it challenging to accurately identify acremonium-like species (Hou et al. 2023) and members of *Scytalidium* studied here. It is noteworthy that although Vu et al. (2019) demonstrated the high efficacy of ITS and LSU in discriminating filamentous fungal species, according to the species delimitation criteria proposed by Jeewon and Hyde (2016), phylogenetic analyses should incorporate ITS and at least one protein-coding gene. However, since most species of *Scytalidium* are represented only by ITS and/or LSU (Table 1), this study, like recent studies on *Scytalidium*, had to rely on a combination of morphological characteristics and phylogenetic analyses based on concatenated ITS and LSU sequences for species identification (Crous et al. 2023; Jeong et al. 2025). This is a limitation, and future work will incorporate secondary barcodes or whole-genome data for phylogenetic reconstruction to further elucidate the taxonomic relationships within *Scytalidium* sensu stricto and sensu lato.

Ecologically, *Scytalidium* has been reported to be associated with plants (wood, plant pathogens, dead branches), basidiomata, soil environments, and insects (Robinson et al. 2007; Kang et al. 2010; Peng et al. 2014; De Medeiros et al. 2019; Crous et al. 2023; Jeong et al. 2025). On one hand, they can cause plant diseases in certain crops, posing ecological threats to agriculture and forestry (Silva et al. 2013). On the other hand, due to their pigment production and various bioactive compounds, they demonstrate potential applications in wood and textile dyeing, as well as other fields (Lapin et al. 2002; Borisova et al. 2015; Hinsch et al. 2022). Additionally, numerous studies have shown that some species of *Scytalidium*, or species previously placed within the genus, are significant human pathogens capable of causing dermatomycoses, respiratory infections, and abscesses (Dickinson et al. 1983; Costa et al. 1988; Moore 1992; Machouart et al. 2013). The primary pathogenic species are *S. hyalinum* and *S. dimidiatum* (which have been merged and are now recognized as *N. hyalinum*; Phillips et al. 2013). Given that *Scytalidium* is a morphological genus, early identifications of clinical isolates based solely on morphological characteristics are questionable. With the incorporation of molecular data, almost all clinical isolates have been identified as either *S. hyalinum* or *S. dimidiatum* (Machouart et al. 2013; Enriquez-Mendez and Gonzalez 2025). Notably, Ringhofer et al. (2025) reported a case of disseminated fungal infection in a dog caused by *S. philadelphianum*. In this study, this species is placed within *Scytalidium* sensu stricto.

In summary, this study has resolved the taxonomic status of most species within *Scytalidium*, although several taxa, including *S. chinense* and *S. japonicum*, remain to be conclusively classified. This will not only facilitate future taxonomic studies of *Scytalidium* and related taxa but also contribute to rapid diagnosis, prevention, and control measures in agricultural, forestry, and clinical settings.

### ﻿A key to accepted species in *Scytalidium*

**Table:** 

1	Sexual and asexual morphs produced	**2**
–	Only asexual morph produced	**3**
2	Ascospores subglobose or globose, > 3 µm in diameter	**4**
–	Ascospores subglobose or globose, 3 µm in diameter	***Scytalidium rodionovae***
3	Arthroconidia not produced from synnemata	**5**
–	Arthroconidia produced from synnemata	***Scytalidium synnematicum***
4	Ascomata 45–165 μm	***Scytalidium ganodermophthorum***
–	Ascomata 25–50 μm	***Scytalidium sphaerosporum***
5	Production of only arthroconidia or chlamydospore-like cells	**6**
–	Simultaneous production of arthroconidia and chlamydospore-like cells	**7**
6	Only arthroconidia produced	***Scytalidium cuboideum***
–	Only chlamydospore-like cells produced	**8**
7	Arthroconidia hyaline to brown	**9**
–	Arthroconidia hyaline or color unknown	**10**
8	Chlamydospore-like cells aseptate	***Scytalidium assmuthi***
–	Chlamydospore-like cells 0–1-septate	**11**
9	Chlamydospore-like cells oval	***Scytalidium terrigenum***
–	Chlamydospore-like cells oblong or ellipsoidal to subglobose	***Scytalidium guizhouense***
10	Arthroconidia longer than 10 μm	***Scytalidium auriculariicola***
–	Arthroconidia shorter than 10 μm	**12**
11	Chlamydospore-like cells oblong to globose, subcylindrical, guttulate or irregular	***Scytalidium chlamydosporum***
–	Chlamydospore-like cells oblong to globose, ellipsoidal	***Scytalidium tongrenense***
12	Chlamydospore-like cells solitary or catenate	**13**
–	Chlamydospore-like catenate	**14**
13	Chlamydospore-like cells intercalary to terminal	***Scytalidium philadelphianum***
–	Chlamydospore-like cells mostly intercalary	**15**
14	Colony on malt agar yellow-red to black	***Scytalidium aurantiacum***
–	Colony on malt agar white to black	***Scytalidium album***
15	Chlamydospore-like cells brown, globose, lobed, or irregularly, 4–9 μm × 3–9 μm	***Scytalidium circinatum***
–	Chlamydospore-like cells dark brown, subglobose to globose, ≤ 7 µm in diameter	***Scytalidium lignicola***

## Supplementary Material

XML Treatment for Scytalidium

XML Treatment for Scytalidium album

XML Treatment for Scytalidium assmuthi

XML Treatment for Scytalidium aurantiacum

XML Treatment for Scytalidium auriculariicola

XML Treatment for Scytalidium chlamydosporum

XML Treatment for Scytalidium circinatum

XML Treatment for Scytalidium cuboideum

XML Treatment for Scytalidium ganodermophthorum

XML Treatment for Scytalidium guizhouense

XML Treatment for Scytalidium lignicola

XML Treatment for Scytalidium philadelphianum

XML Treatment for Scytalidium rodionovae

XML Treatment for Scytalidium sphaerosporum

XML Treatment for Scytalidium synnematicum

XML Treatment for Scytalidium terrigenum

XML Treatment for Scytalidium tongrenense

XML Treatment for Scytalidium xizangensis

XML Treatment for Cladosporium thermophilus

XML Treatment for Cladosporium terminale

XML Treatment for Cladosporium dimorphospora

XML Treatment for Cladosporium infestans

XML Treatment for Caliciopsis uredinicola

XML Treatment for Scytalidium multiseptatum

XML Treatment for Scytalidium tibetense

XML Treatment for Scytalidium japonicum

XML Treatment for Scytalidium chinense

XML Treatment for Scytalidium fulvum

XML Treatment for Scytalidium hepiali

XML Treatment for Scytalidium melanoxylicola

XML Treatment for Scytalidium nielamuense

XML Treatment for Scytalidium verruculosum

XML Treatment for Scytalidium xigazense

## ﻿Additional information

### Use of AI

No use of AI was reported.

### Adherence to national and international regulations

All the fungal strains used in this study have been legally obtained, respecting the Convention on Biological Diversity (Rio Convention).

### Author ORCIDs

Shuo-Qiu Tong https://orcid.org/0009-0006-9422-5551

Yi-Fan Yang https://orcid.org/0009-0004-5897-8236

Peng Li https://orcid.org/0009-0000-9175-6599

Yong-Jun Wu https://orcid.org/0000-0003-0272-6422

Bing-Da Sun https://orcid.org/0000-0003-0272-6422

Zhi-Yuan Zhang https://orcid.org/0000-0003-2031-7518

### Data availability

Sequence data are available in GenBank (see Table 1), and the alignment is provided in the Supplementary material.

## Supplementary materials

Supplementary material 1 Analysis 1 and Analysis 2 Data type zip https://binary.pensoft.net/file/1437686This dataset is made available under the Open Database License (http://opendatacommons.org/licenses/odbl/1.0/). The Open Database License (ODbL) is a license agreement intended to allow users to freely share, modify, and use this Dataset while maintaining this same freedom for others, provided that the original source and author(s) are credited.Shuo-Qiu Tong, Yi-Fan Yang, Peng Li, Yong-Jun Wu, Bing-Da Sun, Zhi-Yuan Zhang
